# 

**Published:** 1887-09

**Authors:** 


### BOOKS RECEIVED

"Anæmia." By Frederick P. Henry, M. D.

"Public Health." The Lamb Prize Essay, 1886. Second edition.

"Maternity, Infancy, Childhood." By John M. Keating, M. D.

"Ligaments, Their Nature and Morphology." By Dr. John Bland Sutton.

"Treatment of Disease in Children." By Angel Money, M. D., M. R. C. P.

"Elementary Microscopical Technology." By Frank L. James, Ph. D., M. D.

"Evacuant Medication, Cathartics, and Emetics." By Henry M. Field, M. D.

"The Physician's Dose and Symptom Book." Seventeenth edition. By Joseph H. Wythe, M. D.

"The Vest-Pocket Anatomist." Thirteenth Revised Edition. By C. Henri Leonard, A. M., M. D.

"A Practical Treatise on Renal Diseases and Urinary Analysis." By William Henry Porter, M. D.

"A Treatise on Diphtheria," etc. By A. Saune Translated by Henry Z. Gill, A. M., M. D., LL. D.

"A Practical Treatise on Diseases of the Eye." By Dr. Edouard Meyer. Translated by Freeland Fergus, M. B.

"Pathology of the Puerperium." Part IV of a Practical Treatise on Obstetrics. By Dr. A. Charpentier. Translated by E. H. Grandin, M. D.

### PAMPHLETS RECEIVED.

"Feeding Patients Against the Appetite." By Ephraim Cutters, M. D., M. M. S.

"A New Treatment of Diseases of the Respiratory Organs and of Blood-Poison, by Rectal Injection of Gases." By Dr. V. Morel.

"The Past, Present and Future Treatment of Homœopathy, Eclecticism and Kindred Delusions." By Henry I. Bowditch, A. M., M. D.

"The Curability of Epilepsy and Epileptoid Affections by Galvanism and Phosphated and Arseniated Bromides." By C. H. Hughes, M. D.

"Some Considerations Concerning Cancer of the Uterus, Especially its Palliative Treatment in its Later Stages. By Andrew F. Currier, M. D.

"A Review of Twenty-two Cottage-Cases, Occurring in the Women's Hospital, in the service of Dr. T. Gaillard Thomas. By H. H. Buckmaster, M. D.

"Congenital Occlusion of the Posterior Nares.' By Alvin A. Hubbell, M. D.

"Transplantation of a Rabbit's Eye into the Human Orbit." By Charles H. May, M. D.

"Healthy Homes and Food for the Working Classes." By Victor C. Vaughan, M. D., Ph. D

"The Physiological Conditions and Sanitary Requirements of School-Houses and School-Life." By A. N. Bell, A. M., M. D.

"Transactions of the Massachusetts Medico-Legal Society," Vol. I, No. 9, 1886.

"Anatomical Researches in the Human Rectum, and a New Method of Rectal Inspection." Part II. By Walter J. Otis, M. D.

"The Doctorate Address Delivered at the Semi-Centennial Anniversary of the University of Louisville, Medical Department." By David W. Yandell, M. D.

C 2cM
LIBRARY INDEXED S.G.O.

Volume LV.] SEPTEMBER, 1887. [Number 3.

### The CHICAGO MEDICAL JOURNAL AND EXAMINER

EDITOR:
S. J. JONES, M.D., LL.D

COLLABORATORS:

CHICAGO:

PROFESSOR E. ANDREWS, M.D., LL.D.
PROFESSOR J. BARTLETT, M.D.
PROFESSOR W. T. BELFIELD, M.D.
PROFESSOR F. BILLINGS, M.D.
PROFESSOR W. H. BYFORD, A.M., M.D.
PROFESSOR L. CURTIS, A.M., M.D.
PROFESSOR N. S. DAVIS, JR., A.M., M.D.
PROFESSOR O. C. DEWOLF, A.M., M.D.
PROFESSOR E. C. DUDLEY, A.B., M.D.
PROFESSOR C. FENGER, M.D.
PROFESSOR J. N. HYDE, A.M., M.D.
PROFESSOR E. F. INGALS, A.M., M.D.
PROFESSOR W. W. JAGGARD, A.M., M.D.
PROFESSOR F. S. JOHNSON, A.M., M.D.
PROFESSOR H. A. JOHNSON, M.D., LL.D.
PROFESSOR C. W. PURDY, M.D.
PROFESSOR C. G. SMITH, A.M., M.D.
PROFESSOR E. WING, A.M., M.D.

MILWAUKEE, WIS.
PROFESSOR N. SENN, M.D.

WASHINGTON, D. C.:
S. O. RICHEY, M.D.

UNITED STATES ARMY:
SURGEON D. L. HUNTINGTON, M.D.

MARINE HOSPITAL SERVICE:
SURGEON S. T. ARMSTRONG, M.D.

UNITED STATES NAVY:
MEDICAL DIRECTOR J. M. BROWNE, M.D.
MEDICAL DIRECTOR A. L. GIHON, A.M., M.D.

